# Maxillary Osteomyelitis with an Incidental Diagnosis of Maxillary Diffuse Large B-Cell Lymphoma: A Case Report

**DOI:** 10.7759/cureus.5238

**Published:** 2019-07-25

**Authors:** Samia Asif, Omar K Abughanimeh, Nedaa M Husainat, Laith Numan

**Affiliations:** 1 Internal Medicine, University of Missouri-Kansas City School of Medicine, Kansas City, USA; 2 Hematology/Oncology, University of Nebraska Medical Center, Omaha, USA; 3 Kidney Institute, University of Kansas Hospital & Medical Center, Kansas City, USA

**Keywords:** osteomyelitis, diffuse large b cell lymphoma, raoultella planticola, maxillary osteomyelitis, incidental findings

## Abstract

Raoultella planticola osteomyelitis is rarely reported in the literature. The most likely source in our case is the oral microbiome secondary to the tooth extraction. Herein we present a case of Raoultella planticola osteomyelitis of the jaw that leads to the diagnosis of diffuse large B-cell lymphoma (DLBCL) of the jaw. A 75-year-old male with no significant medical history, presented to the emergency department with right upper jaw pain after he had a tooth extraction a week before his presentation. Computed tomography (CT) scan of the face showed concerns of right maxillary osteomyelitis with soft tissue swelling and prominent cervical lymph nodes. He underwent a bone biopsy of the maxilla and was started on intravenous ampicillin-sulbactam. His bone culture grew pan-sensitive Raoultella planticola. in addition to that, his bone biopsy revealed diffuse large B-cell lymphoma of the jaw. The patient underwent staging imaging, and he was found to have metastasis to the liver. He was started on chemotherapy and had a good response. In conclusion, Raoultella planticola osteomyelitis is extremely rare. The diagnosis of maxillary DLBCL can be a challenge. Fortunately, our patient had an infection at the same site that led to the diagnosis of DLBCL.

## Introduction

Raoultella planticola is a gram-negative, aerobic bacillus which inhabits natural environments like water, soil, and plants and is not typically known to cause invasive infections in humans [1]. It has been reported to colonize the upper respiratory and gastrointestinal tract. Rarely it can manifest as clinically significant infection, with only a few such cases reported in the literature [2]. Similarly, the involvement of the jaw by non-Hodgkin’s lymphoma (NHL) is very uncommon. Here, we present the first reported case of maxillary osteomyelitis caused by R. planticola with bone pathology, subsequently revealing diffuse large B-cell lymphoma (DLBCL) at the same site. 

## Case presentation

**Figure 1:**
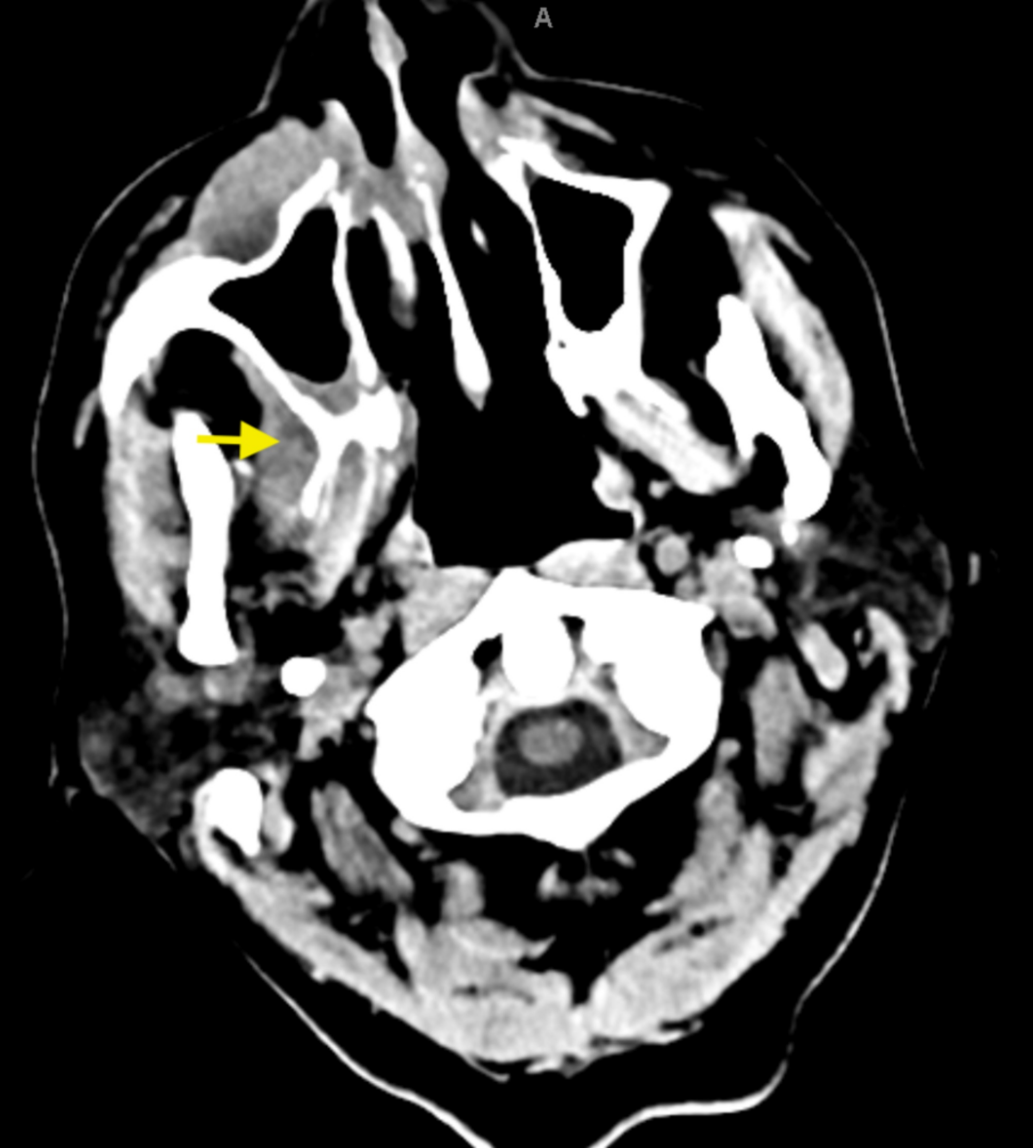
Computed tomography scan of the face showing inflammation, soft tissue swelling, and possible osteomyelitis of the maxilla.

A 75-year-old male with no significant medical history, presented to the emergency department with right upper jaw pain after he had a tooth extraction a week before his presentation. After the tooth extraction, the patient was complaining of worsening pain in the upper jaw, and he started experiencing fever and chills as well. On review of systems, the patient endorsed night sweats and weight loss for the last two months. On physical examination, he was febrile with a temperature of 103 F, the rest of his vital signs were stable. Computed tomography (CT) scan of the face showed concerns of right maxillary osteomyelitis with soft tissue swelling (Figure 1). The patient was admitted for further workup and intravenous antibiotics.

Oromaxillofacial surgery evaluated the patient and took him to the operative room, where he underwent irrigation and debridement, a bone biopsy of the maxilla, and extraction of multiple teeth. Infectious disease team recommended starting the patient on intravenous ampicillin-sulbactam while waiting for the culture results. His bone culture grew pan-sensitive Raoultella planticola. Based on the above findings, the teams put a plan to discharge the patient on levofloxacin and metronidazole to finish six weeks of antibiotics.

**Figure 2:**
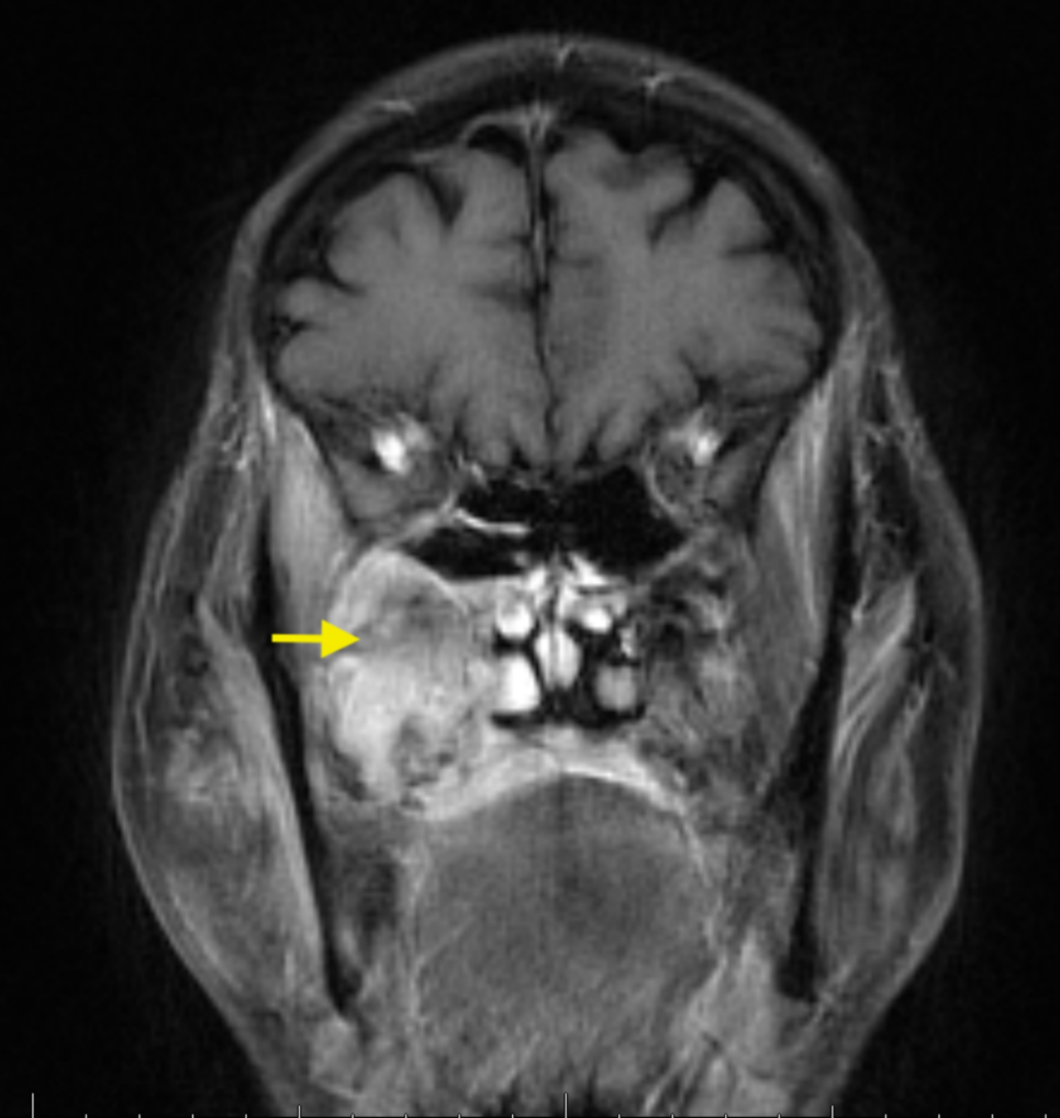
MRI of the face showing large infiltrative soft tissue lesion with extensive surrounding enhancement (DLBCL and superimposed osteomyelitis). MRI - magnetic resonance imaging; DLBCL - diffuse large B-cell lymphoma

**Figure 3:**
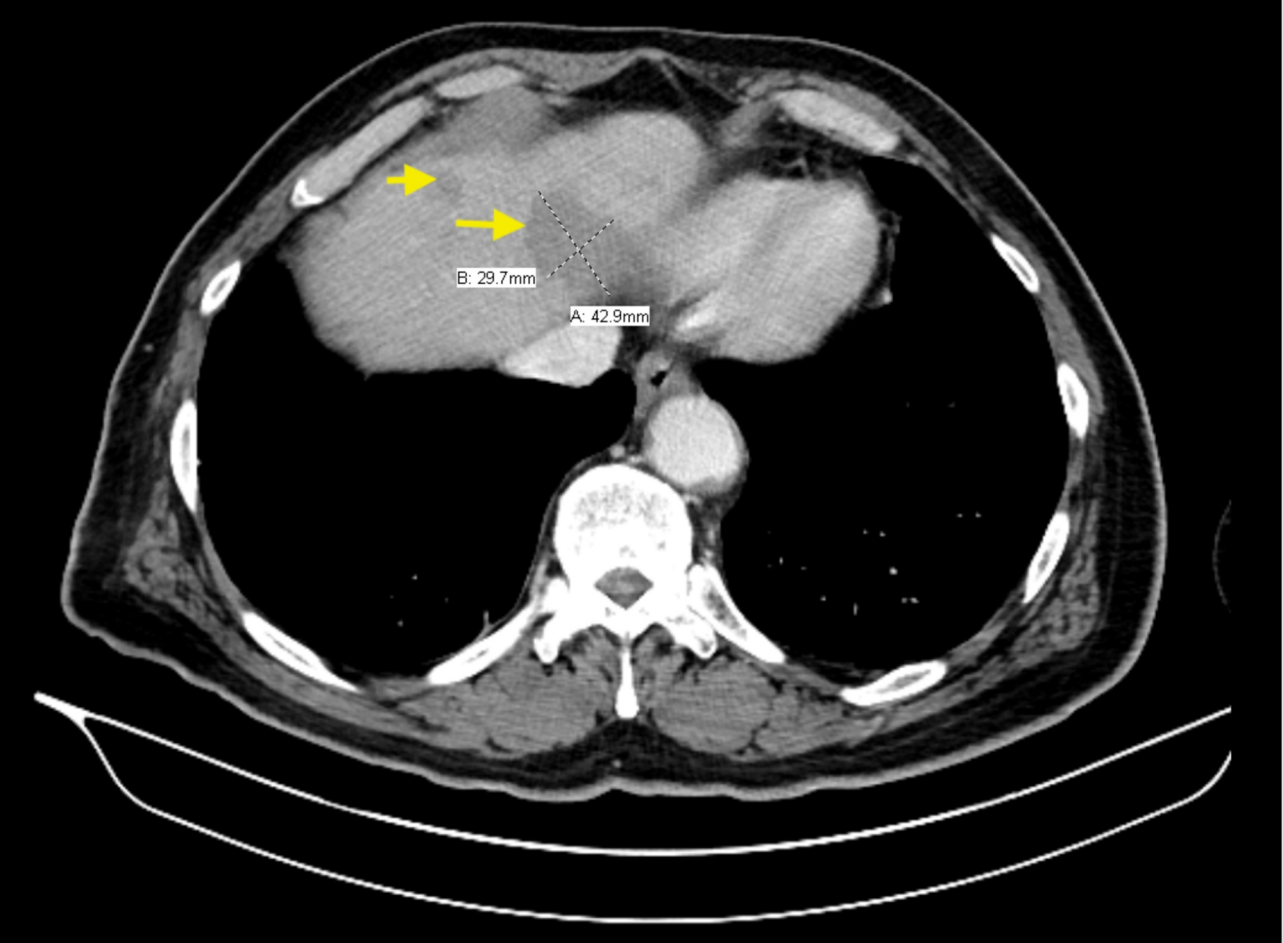
Computed Tomography scan of the abdomen revealing metastasis to the liver.

Surprisingly, his bone biopsy revealed an unexpected finding, as he was found to have diffuse large B-cell lymphoma affecting the same site of the osteomyelitis. Magnetic resonance imaging (MRI) of the face revealed a large infiltrative soft tissue lesion with extensive surrounding enhancement involving the right side of the face, especially the maxilla, and it was extending intracranially (Figure 2). The patient underwent staging imaging, and he was found to have stage IV DLBCL with metastasis to the liver (Figure 3). The oncology team evaluated the patient, and they recommended to start chemotherapy. Eventually, the patient was started on rituximab, cyclophosphamide, doxorubicin, vincristine, and prednisone (R-CHOP) chemotherapy. The patient completed a total of six cycles with minor complications secondary to chemotherapy such as nausea, vomiting, or anemia requiring transfusions. Fortunately, he had an excellent response to chemotherapy, and he continued to follow up with the oncology clinic.

## Discussion

Raoultella species are aerobic, gram-negative bacilli that belong to the family of Enterobacteriaceae. R. planticola and R. ornithinolytica have been identified as causative pathogens in reported cases. R. planticola, previously described as Klebsiella planticola, was re-named in 2001 based on 16S ribosomal RNA and rpoB (i.e., gene encoding the beta subunit of RNA polymerase) gene sequencing [3].

While it is known as a harmless aquatic, botanic, and soil organism, prior studies estimate up to nine to 18% of humans being colonized with R. planticola [4]. Risk factors for colonization of the upper respiratory and digestive tracts include a prolonged stay in intensive care unit (ICU), ongoing chemotherapy, premature birth, diabetes, presence of an enteral feeding tube, long term antibiotic therapy, trauma with soil contamination, invasive procedures such as endoscopies and indwelling urinary catheters [3, 5].

Freny et al. reported the first case of human disease by R. planticola (at the time, named Klebsiella trevisanii) in 1984: a 69-year-old female admitted for infective endocarditis requiring ICU stay and found to have R. planticola bacteremia nine days following mitral valve replacement [6]. A literature search reveals only 22 prior cases of severe infections with R. planticola. The most frequently reported infections included bacteremia, pneumonia, urinary tract infections including cystitis and prostatitis, skin and soft tissue infections such as cellulitis and necrotizing fasciitis and intra-abdominal infections such as cholangitis, cholecystitis, pancreatitis, and gastroenteritis [7]. Only one prior case of osteomyelitis caused by R. planticola has been reported, with diagnostic studies in this case also suggesting the presence of R. ornithinolytica [8]. Only one prior case of osteomyelitis secondary to R. ornithinolytica has been reported previously [8]. In addition to identification by traditional body fluid (blood, urine, sputum, etc.) cultures, determining the specific pathogen may involve using newer techniques such as polymerase chain reaction (PCR), biochemical techniques, VITEK® 2 (BioMérieux, Marcy-l'Étoile, France) or API systems, as previously described by other authors [9, 10].

Most R. planticola isolates in reported cases have been sensitive to third and fourth generation cephalosporins, beta-lactam, and beta-lactamase inhibitor combinations and carbapenems [11]. Recently, nosocomial infections by carbapenem-resistant R. planticola (CRRP) have been reported. The mechanism underlying this resistance involves the production of carbapenemases, including class A beta-lactamase (KPC), class B metal-beta lactamase (IMP-8, NDM-1) and class D beta-lactamase (OXA-48) [7, 11]. In the six reported cases of CRRP so far, patients were noted to have active malignancy (67%) with or without chemotherapy (50%) or neutropenia (33%), and half the cases had received antimicrobial therapy with carbapenems. CRRP infections were associated with poorer outcomes with 67% patients expiring, although this may also be confounded by the fact that these patients had coexisting severe illnesses that affected outcomes too. CRRP, in all cases, was unanimously sensitive to amikacin but variably sensitive to levofloxacin and tigecycline [7].

Non-Hodgkin’s lymphoma comprises two percent of all primary bone tumors [12]. Diffuse large-B cell lymphoma is the most common subtype of NHL [13]. Up to 40% of NHL occurs in extranodal sites, with five percent of these occurring in bones [12, 14]. Of these, two to three percent may occur primarily in the oral cavity and jaws [15]. DLBCL involves the oral cavity in only 0.1% of cases. The maxilla is the most common site of osseous lymphoma in the head and neck region, with the mandible being the second most common site. Presenting symptoms include jaw swelling, dental or jaw pain, and numbness or dysesthesias over the mental region [16]. Tooth mobility and persistent pain and swelling after tooth extraction may be seen. However, establishing the diagnosis of NHL by imaging alone is difficult. Osteolytic and erosive bone features may be seen in most cases, while sclerotic or mixed radiographic appearance may be seen in remaining cases [16]. The primary challenge to diagnosis in these cases is usually a low index of suspicion. If an inadequate bone biopsy specimen is obtained, overlying inflammatory changes may result in an erroneous diagnosis of a reactive or infectious process. An incisional or excisional biopsy of the site of involvement is essential to determine the subtype of NHL in conjunction with immunohistochemistry (IHC) and flow cytometry [13].

Clinical staging, preferably by a PET/CT scan with or without a bone marrow biopsy, allows for differentiation between a primary osseous NHL versus the presence of disseminated disease with bone involvement. Treatment includes chemotherapy with or without radiation therapy depending on disease subtype and stage. For patients with DLBCL who have a partial response or progressive disease following initial chemotherapy, second-line chemotherapy regimens are initiated if the patient is a candidate for autologous hematopoietic stem cell transplantation. Otherwise, palliative involved-site radiation therapy (ISRT), clinical trial enrollment, or supportive care is pursued [13].

## Conclusions

R. planticola is a potential emerging pathogen which may produce serious infections involving different organs systems. The most likely source in our case was the oral microbiome secondary to the tooth extraction. Clinicians should be alert to the possibility of encountering CRRP, which may portend a poorer prognosis. A thorough history and physical examination, together with a low index of suspicion, will help prevent a delayed diagnosis of primary NHL involving bones. Fortunately, our patient had an infection at the same site that led to the diagnosis of DLBCL. Initiating treatment in DLBCL is time-sensitive.
